# An Ultra-Sensitive Monoclonal Antibody-Based Competitive Enzyme Immunoassay for Sterigmatocystin in Cereal and Oil Products

**DOI:** 10.1371/journal.pone.0106415

**Published:** 2014-09-03

**Authors:** Min Li, Peiwu Li, Hui Wu, Qi Zhang, Fei Ma, Zhaowei Zhang, Xiaoxia Ding, Hengling Wang

**Affiliations:** 1 Oil Crops Research Institute of the Chinese Academy of Agricultural Sciences, Wuhan, P. R. China; 2 Key Laboratory of Biology and Genetic Improvement of Oil Crops, Ministry of Agriculture, Wuhan, P. R. China; 3 Key Laboratory of Detection for Mycotoxins, Ministry of Agriculture, Wuhan, P. R. China; 4 Laboratory of Risk Assessment for Oilseeds Products (Wuhan), Ministry of Agriculture, Wuhan, P. R. China; 5 Quality Inspection and Test Center for Oilseeds Products, Ministry of Agriculture, Wuhan, P. R. China; University of Nottingham, United Kingdom

## Abstract

Sterigmatocystin (STG), a biosynthesis precursor of aflatoxin B_1_, is well known for its toxic and carcinogenic effects in humans and animals. STG derivatives and protein conjugates are needed for generation of monoclonal antibodies (mAbs). This work describes a reliable and fast synthesis of novel STG derivatives, based on which novel STG bovine serum albumin conjugates were prepared. With the novel STG bovine serum albumin conjugates, three sensitive and specific mAbs against STG, named VerA 3, VerA 4, and VerA 6, were prepared by semi-solid hypoxanthine/aminopterin/thymidine (HAT) medium using a modified two-step screening procedure. They exhibited high affinity for STG and no cross-reactivity (CR) with aflatoxins B_1_, B_2_, G_1_, G_2_, and M_1_. Based on the most sensitive antibody VerA 3, an ultra-sensitive competitive enzyme-linked immunosorbent assay (ELISA) was developed for STG in wheat, maize, and peanuts. Assays were performed in the STG-GA-BSA-coated (0.5 µg·mL^−1^) ELISA format, in which the antibody was diluted to 1∶80,000. Several physicochemical factors influencing assay performance, such as pH, ionic strength, blocking solution, and diluting solution, were optimized. The final results showed that the assays had the detection limits of 0.08 ng·g^−1^ for wheat, 0.06 ng·g^−1^ for maize, and 0.1 ng·g^−1^ for peanuts, inter-assay and intra-assay variations of less than 10%, and recoveries ranging from 83% to 110%. These recoveries were in good agreement with those obtained by using HPLC-MS/MS method (90–104%), indicating the importance of the mAb VerA 3 in the study of STG in crude agricultural products.

## Introduction

Sterigmatocystin (STG) is toxic, mutagenic, and carcinogenic secondary metabolites primarily produced by Aspergillus species such as Aspergillus versicolor, Aspergillus flavus, Aspergillus nidulans, and Aspergillus rugulosus [1], [2]. Sterigmatocystin can contaminate many types of food and feed, especially wheat, maize, peanuts, and forage [3]. As a biosynthesis precursor of aflatoxin B_1_, sterigmatocystin has a common structure containing furan rings and xanthones similar with aflatoxin B_1_ [4]. The toxicity of sterigmatocystin is only second to aflatoxin B_1_, which seriously threatens human and animal health [1], [3]–[5]. To protect the agricultural environment, assess quality of commercial agro-products and foods, and safeguard health and lives of consumers, a few countries have established maximum limits of sterigmatocystin in agro-products. For example, Czech Republic and Slovakia have set regulations on STG in rice, vegetables, potatoes, flour, poultry, meat, and milk at a level of 5 µg·kg^−1^, and in other foods at a level of 20 µg·kg^−1^ [1]. However, the regulatory sterigmatocystin level of below 25 µg·kg^−1^ is accepted in China [6]. Therefore, detecting sterigmatocystin in grains is crucial.

Several well-established methodologies for analyzing aflatoxins in different foods have been reported, such as thin layer chromatography (TLC), high performance liquid chromatography (HPLC) with fluorescence detection or mass spectrometry [5], [7]. To date, the most frequently applied analytical methods are based on TLC due to its simplicity, but it lacks sensitivity and accuracy [8]. In contrast, chromatographic methods including HPLC and mass spectrometry demonstrate high sensitivity and accuracy, but require extensive sample preparation, expensive equipment, and well-trained personnel [7], [9]. Recently, enzyme-linked immunosorbent assay (ELISA) methods mainly used for routine analysis have also been proposed, which are usually simple, portable, and reliable for the analysis of a large number of samples compared to chromatographic techniques [9]. Currently, although indirect competitive ELISA (icELISA) is commonly used for mycotoxin analysis, the so-called matrix effect or matrix interference that is common in ELISA methods will result in underestimates or overestimates of the mycotoxin concentrations in product samples [10]. Therefore, inclusion of a sample cleanup step could further increase the reliability of the assay [11].

MAb, the key reagent for immunoassay, plays an important role in icELISA for STG analysis. However, STG derivatives and protein conjugates are needed for generation of monoclonal antibodies (mAbs). In this work, we present a simple, fast and reliable synthesis for novel STG derivatives and STG bovine serum albumin conjugates. Based on the novel STG bovine serum albumin conjugates, we herein obtained three sensitive and specific monoclonal antibodies (mAbs) against sterigmatocystin, which have advantages of consistency, constant properties, and unlimited production. With these mAbs, an immunoaffinity column (IAC) was developed and employed for sample preparation. Meanwhile, a sensitive icELISA method was developed, optimized, and validated for detecting sterigmatocystin in wheat, maize, and peanuts.

## Material and Methods

This study was carried out in strict accordance with the recommendations in the Guide for the Care and Use of Laboratory Animals of the National Institutes of Health. The protocol was approved by the Laboratory Animal Monitoring Committee of Hubei Province and performed accordingly. All surgery was performed under sodium pentobarbital anesthesia, and all efforts were made to minimize suffering. The statistical analysis was conducted by Origin 8.0.

### Chemicals and instruments

Sterigmatocystin, aflatoxins B_1_ (AFB_1_), B_2_ (AFB_2_), G_1_ (AFG_1_), G_2_ (AFG_2_), and M_1_ (AFM_1_), glycolic acid (H_2_O ∼1%), dioxane (H_2_O ≤0.01%), goat anti-mouse immunoglobulin horseradish peroxidase (IgG–HRP), mouse monoclonal antibody ISO2-1 kits, BSA (≥98%, agarose gel electrophoresis grade, art. no. A3675), complete Freund's adjuvants (CFA), incomplete Freund's adjuvants (IFA), urea-hydrogen peroxide (97%), 3, 3′, 5, 5′-tetramethylbenzidine (TMB), hypoxanthine/aminopterin/thymidine (HAT), hypoxanthine/thymidine (HT), and polyethylene glycol 1450 (PEG 1450, 50%) were purchased from Sigma-Aldrich (St. Louis, MO). N-hydroxysuccinimide (NHS) and N, N-dicyclohexylcarbodiimide (DCC) were purchased from Fluka. RPMI-1640 medium with l-glutamine and HEPES (free acid, 283.3 g/L) were obtained from HyClone. Fetal bovine serum, penicillin (+10,000 U/mL), and streptomycin (+10,000 µg/mL) were from Gibco. Unless otherwise stated, all other inorganic chemicals and organic solvents were of analytical-reagent grade or better. Water was obtained from a MilliQ purification system (Millipore).

### Animals and cells

Six-week-old female BALB/c mice were purchased from Disease Control and Prevention Center of Hubei Province (Wuhan, China). SP2/0 myeloma cells were purchased from China Center for Type Culture Collection (CCTCC, Wuhan, China).

### Synthesis of novel immunogens

**Figure 1:**
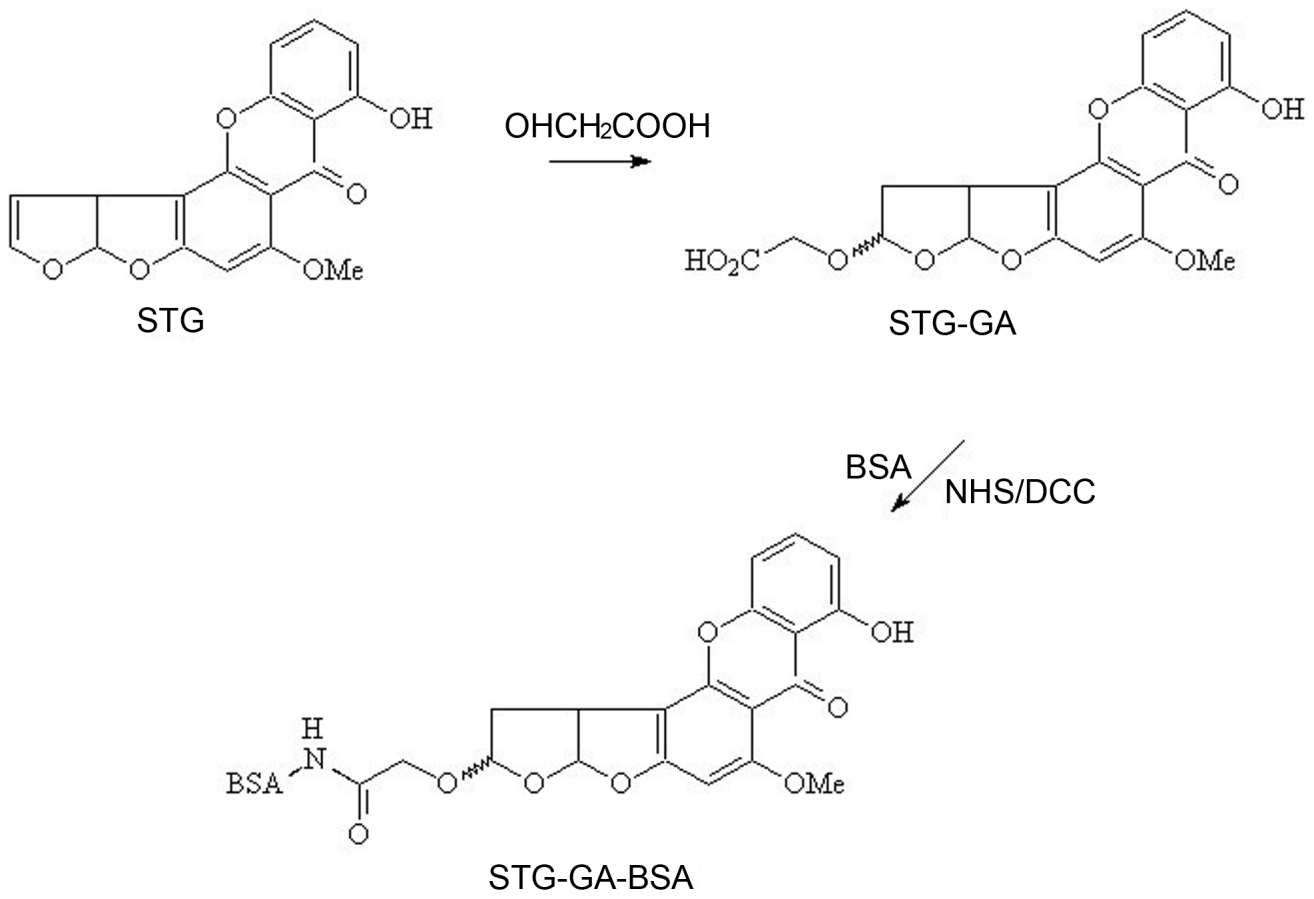
Synthesis procedure for the sterigmatocystin artificial antigen through AEM.

Antigens were prepared following the method as described by Cervino et al. with some modifications [12]. In a heated 10 mL flask with a septum and stirring bar, 0.5 g glycolic acid was dissolved in 4 mL dry trifluoroacetic acid (TFA). Five micrograms of STG were dissolved in 2 mL acetonitrile, this fresh solution of STG was added to the glycolic acid/TFA mixture with a syringe, and the reaction was allowed to proceed for 2 h at room temperature with vigorous stirring. Then, the organic solution was removed with a rotary evaporator under reduced pressure and the synthesized product STG-GA was obtained. An optimized active ester method (AEM) was employed to synthesize artificial antigens of sterigmatocystin. Briefly, all of the STG-GA hapten was suspended in 0.4 mL dioxane, and then 8 mg N-hydroxysuccinimide (NHS) was added and reacted for 1 h at room temperature. Fifteen milligram dicyclohexylcarbodiimide (DCC) was added to the solution and reacted for 4 h at room temperature. The mixture was settled down during the subsequent 24 h incubation period in a dark chamber. After centrifuged at 8,000 r/min for 5 min, the supernatant was added dropwise to 9.6 mg of the BSA dissolved in 5 mL 0.2 mol·L^−1^ PBS (pH 8.0) and kept at room temperature. The produced mixture was stirred by a rotor for 4 h in a dark chamber at room temperature. After centrifuged (3,000 r/min, 10 min), the obtained supernatant was dialyzed with 0.01 mol/L PBS at pH 8.0 (3 days, 6 h/time) and followed by phosphate buffer (PB) for 2 days (8 h/time). After dialyzing, the artificial antigen sterigmatocystin-GA-BSA (STG-GA-BSA) was stored at −20°C until further use. The synthetic route for the sterigmatocystin complete antigen is shown in Fig. 1.

### Immunization

In the initial immunization, 200 µg STG-GA-BSA conjugate was dissolved in a sterilized 0.9% NaCl solution and then emulsified with an equal volume of CFA. Four six-week-old female BALB/c mice were immunized by multiple-point subcutaneous injection of the final water-in-oil emulsion detailed above. Booster injections were given three times at 3-week intervals, with an equal volume of the antigen emulsified with IFAs. To check the immune response to immunogen, the antisera were assayed for anti-sterigmatocystin antibodies by indirect ELISA nine days after the antisera were collected from the tails of the four mice. After the fourth immunization, the antisera from the four mice were determined by icELISA. By comparison, the mouse with higher sensitivity and better cross-reactivity (CR) with four aflatoxins (aflatoxins B_1_, B_2_, G_1_ and G_2_) was sacrificed for hybridoma production after booster injection, which used a double dose of antigen dissolved in the PBS without emulsification with adjuvant.

### Cell fusion and screening

For hybridoma production, the spleen of the mouse was removed aseptically and the splenocytes were isolated. Spleen cells (1×10^8^) were fused with freshly isolated SP2/0 myeloma cells (2.2×10^7^) according to the method described elsewhere in presence of 50% polyethylene glycol (PEG 1450) [13]. Then, the fusion cells were suspended with the complete medium containing high-fructose corn syrup (HFCS) and equally distributed into six 96-well plates before incubated in 5% CO_2_ at 37°C. The screening of the fused cells was performed by liquid culture, and the supernatants were tested in a two-step screening procedure.

STG-GA-BSA mainly consists of STG and BSA. When STG-GA-BSA was injected into the animals, abundant anti-BSA antibodies were secreted from serum along with the production of anti-STG antibodies. To address this issue, BSA was added into the serum to eliminate the antibodies against BSA before STG-GA-BSA was used as coating antigen in ELISA.

### Indirect noncompetitive and competitive ELISA

In indirect noncompetitive ELISA (incELISA), the plates were coated with 100 µL/well STG-GA-BSA at an appropriate concentration in 0.05 M carbonate-bicarbonate buffer (pH 9.6) and sustained for 2 h at 37°C. After washing three times with 300 µL 0.05% PBST, 200 µL of 1.5% OVA in the PBST solution was added to each well and incubated for 1 h at 37°C. After other three washing steps, 100 µL/well mAbs with an appropriate dilution were added into each well of the plates. After 1 h incubation at 37°C, the plates were rewashed, IgG–HRP (diluted at 1/5000 in the PBST, 100 µL/well) was added, and then the plates were incubated for 45 min at 37°C. After sixfold washing, the color was developed by adding 100 µL freshly prepared substrate solution (composed of 9.5 mL pH 5.0 phosphate-citrate buffer, 0.5 mL 2 mg/mL TMB (dissolved by ethanol), and 32 µL 3% (w/v) urea-hydrogen peroxide), and the mixture was incubated for 15 min at 37°C in the dark. Then, 50 µL of the stop solution (2 M H_2_SO_4_) was added to each well and the absorbance at 450 nm was measured with a microplate reader.

IcELISA was carried out to determine antibody sensitivity of mouse sera or cell culture supernatants. The procedure was identical with that of incELISA except that 50 µL/well of mAb diluted in PBST and 50 µL/well of analyte dissolved in 10% methanol–PBS were added after blocking. Sigmoidal curves were fitted to a logistic equation from which IC_50_, IC_20_, IC_80_ values (concentrations at which binding of the antibody to the coating antigen are inhibited by 50%, 80% and 20%, respectively) and recoveries were determined.

### Production and characterization of antibodies

Ascitic fluids were produced by inoculating hybridoma cells into IFA-treated BALB/c mice. The IgG fractions were prepared by caprylic acid-ammonium sulfate precipitation.

To assess the titers of the antibodies for each of the secreted hybridoma cell strains, incELISA was performed as described above. Subsequently, icELISA was used to evaluate the sensitivity of each type of mAb as previously described. In addition to sterigmatocystin, cross-reactivity (CR) with major aflatoxins (AFM_1_, AFB_1_, AFB_2_, AFG_1_, and AFG_2_) was carried out. Based on these data, a plot of antibody inhibition expressed as a percentage of B/B_0_ was drawn, where B was the absorption at each concentration of the analyte and B_0_ was the absorbance in the absence of analyte. CR for different aflatoxins was determined by comparing the half-maximal inhibitory concentrations (IC_50_) of the analyte and calculated as: CR  =  (IC_50 STG_/IC_50 analyte_) ×100%. The affinities of the antibodies were determined by incELISA.

The isotype classification of the mAbs was performed with a commercially available ISO2-1 kit according to the protocol provided by the kit manufacturer.

### Optimization of icELISA

The serial concentrations of coating antigen were prepared by appropriate dilutions of STG-GA-BSA from 2 ng·mL^−1^ to 0.125 ng·mL^−1^ with a dilution factor of 2 in the carbonate-bicarbonate buffer using the checkerboard procedure.

IcELISA was optimized as previously reported (Guan et al., 2011) with some modifications [9]. In these assays, sterigmatocystin was used as the competitor analyte and IC_50_ as the main criterion to evaluate immunoassay performance. To improve the sensitivity of icELISA, four experimental parameters, including pH, blocking solution, STG dilution, and ionic strength, were studied sequentially. The PBST solutions with different pH values (ranging from 5 to 9) were first evaluated. The effects of blocking reagents (1% OVA, 1% BSA and 1.5%skim milk powder) were then investigated. Thirdly, STG-dilute solutions (10, 20, 30, and 40% methanol–PBS (v/v)) were studied. And finally, PBST with different salt concentrations was determined to investigate the effect of ionic strength.

### Preparation and characterization of the immunoaffinity column (IAC)

To eliminate the influence of impurities generated in sample extraction on ELISA and improve the sensitivity of this method, the immunoaffinity column (IAC) was prepared as described by Wang et al. with some modifications [14]. The procedures were as follows: 10 µg CNBr-activated Sepharose-4B matrix powder was dissolved into 1 mM HCl and then placed into a sintered-glass filter funnel and washed with the HCl solution for 15 min; the CNBr-activated Sepharose-4B was sequentially washed with 25 mL coupling buffer for 5 times, and then quickly transferred into the coupling buffer with 5 mg mAb and thoroughly mixed by stirring (180 rpm for 1 min) at room temperature for 2.5 h; the coupling products were obtained with a sintered-glass filter funnel and washed with the coupling buffer and blocking buffer to remove free ligands, and then transferred into 0.1 M Tris-HCl buffer (pH 8.0) and stirred (180 rpm for 1 min) at room temperature for 2 h. To remove the uncoupled blocking ligands, the coupling products were sequentially washed with 0.1 M HAc-NaAc (pH 4.0) and Tris-HCl buffer for at least 5 cycles. The coupling products were washed with 5 times the volume of 0.01 M PBS to prepare suspension and stored in 0.01 M PBS containing 0.01% NaN_3_ solution at 4°C.

For dynamic affinity capacity characterization of the IAC, 200 ng STG dissolved in 10 mL methanol–PBS (10%, v/v) passed through the IAC at a flow rate of 1 mL/min. The saturated column was washed with 5 mL pure water and then eluted with 1 mL methanol. The eluate was collected and determined by HPLC, and in this way, the amount of STG in the eluate was used to indicate the capacity of the column. In the evaluation of the recovery of IAC, 10 mL of STG standard solution in two different concentrations (5 and 50 ng·mL^−1^) was loaded onto the columns the same procedure as capacity identification. Every identification was studied in triplicate.

### IcELISA analysis for spiked samples

In this work, the spiked wheat, maize, and peanut samples were prepared as reported. The STG-free samples were finely ground, and 5 g sample was spiked with STG at concentrations of 5, 10, and 20 ng·g^−1^. Then, the 5 g spiked sample was extracted with 25 mL methanol–water (8∶2, v/v) by vortex mixing for 3 min, was held for at least 5 min, and then filtered through two-double filter paper. The clear extract (2 mL) was diluted with 4 mL water and filtered through 0.45- µm filter membrane. The diluted filtrate was cleaned up and concentrated through the IAC. Subsequently, the IAC was eluted with 1 mL methanol at a flow rate of about one drop per second. The resulting eluate was evaporated to dryness under nitrogen at 60°C, and then redissolved in 4 mL methanol–PBS (1∶9, v/v). The eluent was used for icELISA analysis. The calibration curves were established by spiking the blank matrix with the standard and processed in the same way as the samples.

### HPLC-MS/MS analysis for spiked samples

The spiked wheat, maize, and peanut samples were prepared as described by Aleksandrs for LC-MS/MS analysis [5]. 5 g sample was spiked with STG at concentrations of 5, 10, and 20 ng·g^−1^, and extracted with 25 mL acetonitrile–water (84∶16, v/v) by vortex mixing for 30 min, and then filtered through double filter paper. The raw extract was diluted with pure water and purified using Strata X (500 mg) SPE column. Subsequently, SPE column was washed with acetonitrile–water (4∶6, v/v), then with methanol–water (4∶6, v/v) and STG was eluted with 4 mL acetonitrile. The resulting eluate was evaporated to dryness under nitrogen at 60°C and redissolved in 1 mL acetonitrile–water (3∶1, v/v). The calibrants were prepared by spiking the blank matrix with the standard and prepared in the same way as the samples.

## Results and Discussion

### Immunization

The STG-GA-BSA conjugate induced all of the six-week-old BALB/c mice to produce hapten-specific antibodies nine days after the initial immunization. With the increase of inoculation time, the titers of the antibodies were enhanced. On the 93rd day, three of the four tested mice gave high antibody titers of more than 10,000 and high sensitivity (data not shown). With higher sensitivity and better cross-reactivity, 4#, 1#, and 2# mice were successively chosen for B-lymphocyte donors for further fusion experiments.

### Cell fusion and screening

Since STG-GA-BSA is conjugated with the carrier protein, it produced antibodies against the carrier and mixtures of these molecules along with the production of antibodies against this small molecular hapten. Therefore, during the immunization and screening procedures, 1% BSA in PBS (m/v) was added into the serum supernatants to react with the anti-BSA antibody and inhibit its combination with coating antigen. Then, STG-GA-BSA was chosen as coating antigen.

In this work, a two-step screening procedure reported by Li and Guan was used to select sensitive and specific mAbs against STG. In total, 658 isolated clones visible to the naked eye were moved into 96-well plates. The wells with high cell density were tested by incELISA, and then 7 positive hybridomas were selected in the first screening procedure. Finally, three stable clones named VerA 3, VerA 4, and VerA 6 were picked out in the second procedure. The results of the three screened clones are shown in Table 1.

**Table 1:** Final screening results of hybridoma cells resulted from fusions of splenocytes.

Clone	Titer^a^ of supernatants	OD 450 nm values		Blank
		STG^b^		
		100 ng/mL	0 ng/mL	
VerA 3	800	0.1012	1.053	0.0873
VerA 4	400	0.1123	1.032	0.0972
VerA 6	50	0.1240	0.986	0.0108

a The titer was defined as the reciprocal of the dilution that gave an absorbance most close to 1.0.

b STG was prepared by diluting the stock solution with 10% methanol-PBS to 200 ng mL^−1^ and mixed with equivalent supernatants, and the final concentrations were 100 ng mL^−1^. Aflatoxins of 0 ng mL^−1^ were the PBS containing the same methanol content.

### Characterization of antibodies

Three stable hybridoma lines VerA 3, VerA 4, and VerA 6 were injected into BALB/c mice, and 5–10 mL ascitic fluid was collected from each mouse. All of the three mAbs had no cross-reactivity with BSA. The affinity constants for VerA 3, VerA 4, and VerA 6 were determined by non-competitive ELISA to be 1.91×10^9^, 2.74×10^8^, and 1.45×10^8^ L/mol, respectively. The titration and isotype determination results are shown in Table 2.

**Table 2:** Titers and isotypes of ascites antibodies.

Clone	Titer^a^	OD 450 nm	Value		Isotype	
		Negative control serum	1% BSA	Blank	Heavychain	Lightchain
VerA 3	6.4×10^5^	0.093	0.091	0.088	IgG1	Kappa
VerA 4	3.2×10^5^	0.106	0.102	0.097	IgG2a	Kappa
VerA 6	3.2×10^5^	0.097	0.089	0.095	IgG2a	Kappa

a The titer was defined as the reciprocal of the dilution that gave an absorbance most close to 1.0.

### Optimization of icELISA

To improve the sensitivity of mAb in icELISA, the effect originated from different physicochemical factors on ELISA was identified, and four experimental parameters influencing assay performance (pH, ionic strength, blocking solution, and STG dilution) were optimized. Before parameter optimization, a satisfying compromise between the lowest detection limit and the minimum reagent expense was obtained by using 0.5 µg·mL^−1^ STG-GA-BSA and 1∶80,000 dilution of the antibody with the checkerboard procedure.

**Figure 2:**
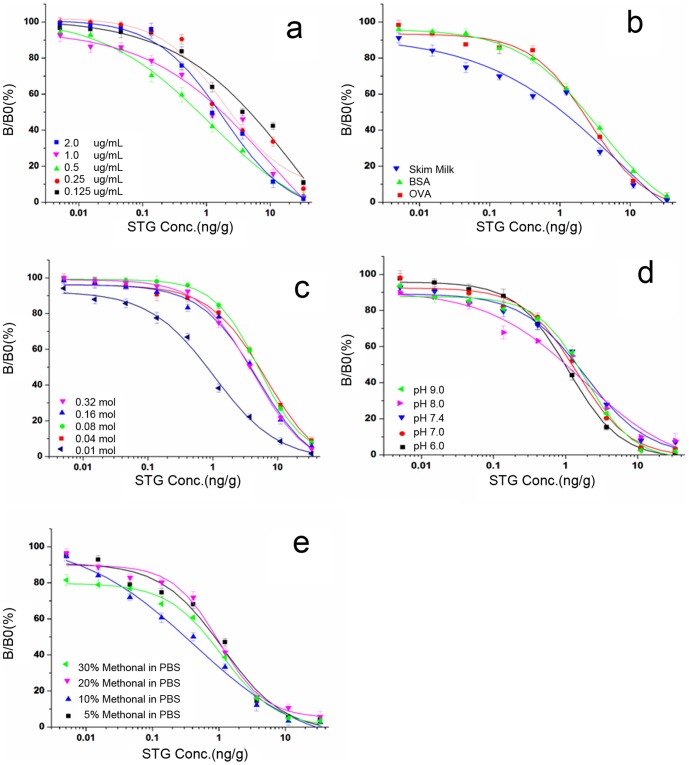
Influences of different factors, including coating antigen and antibody dilution ratio (a), blocking reagents (b), pH (c), ionic strength (d), and diluting solution (e) on the performance of the assay, with the results being the mean of three independent experiments.

pH is one of the main factors influencing the assay performance. Fig. 2d indicated that a slightly acid solution resulted in a relatively lower IC_50_ value, so the value of pH 6.0 was selected as the optimum for the assay. Ionic strength influenced the ELISA performance with the optimum concentration of 0.01 M (Fig. 2c), which led to the lowest IC_50_ value. The effects of blocking reagents (1% OVA, 1% BSA and 1.5% skim milk powder) were investigated because they were usually used in the ELISA system to eliminate non-specific binding. Finally, 1.5% skimmed milk with the lowest IC_50_ value was selected (Fig. 2b). Since buffer media may affect the antibody–antigen reaction, different dilute solutions with multiple concentrations of organic solvents were evaluated, and the best STG dilution was determined to be 10% methanol–PBS (v/v) (Fig. 2e).

By studying the preceding factors, the optimum conditions of the icELISA procedure were obtained: the blocking reagent was 1.5% skim milk, pH was 6.0, ionic strength was 0.01 M, and STG dilution was 10% methanol–PBS (v/v).

### Sensitivity and specificity of antibodies

The titers and sensitivity of the antibodies against STG were analyzed by optimized ELISA. The results in Table 3 showed that VerA 3 had the highest sensitivity, so VerA 3 was selected for further evaluation. The detection limit (IC_10_) and sensitivity (IC_50_) of VerA 3 towards STG were 0.005 ng·mL^−1^ and 0.36 ng·mL^−1^, respectively. The linear working range (IC_20_–IC_80_) was 0.03–2.7 ng·mL^−1^. The cross-reactivity results demonstrated that VerA 3 had no cross-reactivity with AFB_1_, AFB_2_, AFG_1_, AFG_2_, and AFM_1_. Being a sensitive and specific antibody, VerA 3 can be utilized to detect STG in multiple types of mycotoxin-contaminated agro-products.

**Table 3:** Sensitivity (expressed as ng mL^−1^) and the minimal and maximal inhibition values of the three monoclonal antibodies.

Clone	Titers		STG(ng/mL)	
		IC_20_ a	IC_50_ b	IC_80_ c
VerA 3	8×10^4^	0.03	0.36	2.70
VerA 4	4×10^4^	0.12	0.75	3.16
VerA 6	4×10^4^	0.098	1.71	16.76

a Concentration at which the binding of the antibody to the coating antigen is inhibited by 20%.

b Concentration at which the binding of the antibody to the coating antigen is inhibited by 50%.

c Concentration at which the binding of the antibody to the coating antigen is inhibited by 80%.

### Characterization of the IAC

The IAC is commonly used for sample preparation and employed in several official methods according to sample cleaning protocols. However, the IAC used for STG determination has not been reported due to lack of sensitive antibodies. In this study, the specific mAb VerA 3 was utilized to prepare the IAC to alleviate the effects of sample matrix on STG determination. The IAC was characterized to check the reliability of this Sepharose-based IAC. To evaluate the maximum capacity of the IAC, the binding capacity was determined by overloading the column with 10 mL of STG standard solution (20 ng·mL^−1^). The evaluation results showed that the columns containing 0.3 mL immunosorbent showed a binding capacity of 160±2 ng (n = 3). The average recoveries for 5 ng·mL^−1^ and 50 ng·mL^−1^ STG were 91.6% and 94.2%, with relative standard deviations of 4.3% and 3.1%, respectively. With good performance in recovery and capacity, the IAC was utilized to prepare samples.

### Establishment of standard curves

**Figure 3:**
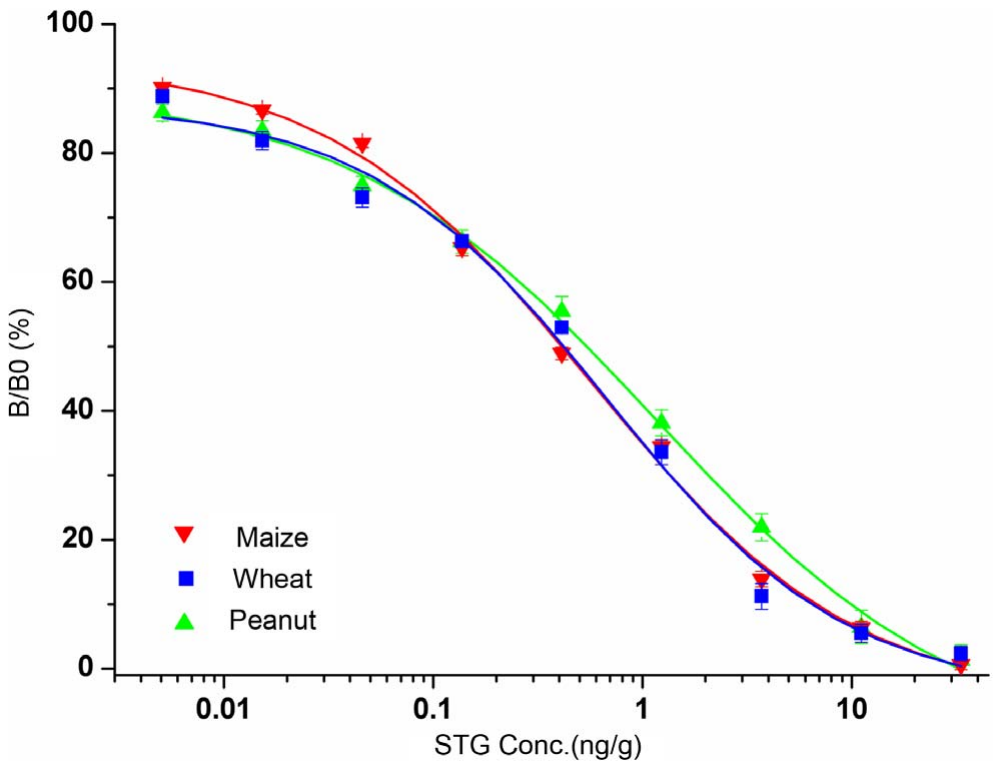
Standard curves for STG with each point representing the mean ± SD from five determinations in competitive ELISA.

To evaluate matrix effects, the calibration curves produced from either pure solvent or STG-free cereals and oil sample extracts were compared, and a significant matrix effect was observed. The calibration curves in cereals and oil products were influenced by the new environment. Therefore, wheat, maize, and peanut matrices were selected to prepare calibration curves (Fig. 3). The IC_50_ values were 0.39 ng·g^−1^ for wheat, 0.38 ng·g^−1^ for maize, and 0.47 ng·g^−1^ for peanuts. The detection limits, defined as the concentrations corresponding to 80% of B/B_0_, were 0.08 ng·g^−1^ for wheat, 0.06 ng·g^−1^ for maize, and 0.1 ng·g^−1^ for peanuts.

### IAC-icELISA of spiked samples

The samples spiked with the STG standards were detected by IAC-icELISA. The intra-assay and inter-assay precision evaluations were performed in six replicates on the same day and in five different days, respectively. The mean recoveries and standard deviations were calculated at each theoretical concentration and summarized in Table 4, which showed that the average recoveries of IAC-icELISA ranged from 83% to 110%. The high recoveries of IAC-icELISA indicated that IAC could specially retain the target analyte, effectively alleviate the matrix effect, and obviously increase the accuracy of icELISA. The developed IAC-icELISA exhibited approximate and acceptable recoveries for different samples, including wheat, maize, and peanuts.

**Table 4:** Recovery analysis of the STG spiked in cereal and oil products.

Matrix		Expected(ng/g)	IAC-icELISA Found^a^ (ng/g)	Recovery (%)	HPLC-MS/MS Found^a^ (ng/g)	Recovery (%)
Wheat	Within assay^b^ (n = 6)	5	5.40±0.40	108.0	4.51±0.21	90.2
		10	9.62±0.45	96.2	9.67±0.35	96.7
		20	18.90±0.78	94.5	20.06±0.64	100.3
	Between assay^c^ (n = 6)	5	5.51±0.53	110.2	4.57±0.08	91.4
		10	9.46±0.61	94.6	9.56±0.04	95.6
		20	19.40±0.97	97.0	20.64±0.13	103.2
Maize	Within assay^b^ (n = 6)	5	4.61±0.37	92.2	4.59±0.18	91.8
		10	10.2±0.51	102.8	9.69±0.27	96.9
		20	20.06±0.74	100.3	20.48±0.34	102.4
	Between assay^c^ (n = 6)	5	4.53±0.41	90.6	4.63±0.11	92.6
		10	9.71±0.89	97.1	9.87±0.08	98.7
		20	19.85±1.04	99.3	20.88±0.20	104.4
Peanut	Within assay^b^ (n = 6)	5	4.17±0.18	83.4	4.55±0.13	91.0
		10	8.74±0.51	87.4	9.33±0.25	93.3
		20	18.65±1.21	93.3	18.92±0.17	94.6
	Between assay^c^ (n = 6)	5	4.39±0.30	87.8	4.52±0.09	90.4
		10	9.21±0.75	92.1	9.25±0.14	92.5
		20	20.14±1.67	100.7	18.88±0.08	94.4

a The reported data are the mean ± SD.

b The assays are carried out in six replicates on the same day.

c The assays are carried out in six different days.

### Validation of the Assay with HPLC-MS/MS

The spiked samples were analyzed with HPLC –MS/MS and results were compared with those using the developed IAC-icELISA. The intra-assay and inter-assay precision evaluations were performed in six replicates on the same day and in five different days, respectively. The results (Table 4) indicated that these results achieved by IAC-icELISA were in good agreement with those obtained using HPLC-MS/MS method. It can be concluded that the competitive ELISA based on mAb VerA 3 demonstrated reliable reproducibility for analyzing STG in cereal and oil products.

## Conclusion

For the determination of STG, three sensitive and specific monoclonal antibodies (mAbs) against STG were identified. They exhibited high affinity for STG and no cross-reactivity with aflatoxins B_1_, B_2_, G_1_, G_2_, and M_1_. IcELISA was developed for the detection of STG by determining the optimal STG-GA-BSA coating antigen and mAb using a checkerboard fashion and competitive ELISA, which were defined to be 0.5 µg·mL^−1^ and a dilution of 1∶80,000, respectively. The effects of the influencing factors such as pH, ionic strength, blocking solution, and diluting solution were verified on the performance of ELISA. The IC_50_ values of 0.39 ng·g^−1^ for wheat, 0.38 ng·g^−1^ for maize, and 0.47 ng·g^−1^ for peanuts, the detection limits of 0.08 ng·g^−1^ for wheat, 0.06 ng·g^−1^ for maize, and 0.1 ng·g^−1^ for peanuts, inter-assay and intra-assay variations of less than 10%, and the recoveries ranging from 83% to 110% were obtained. According to the analysis of spiked samples, the results were in good accordance with the HPLC-MS/MS method. in conclusion, this new, sensitive, and specific mAb could be used to develop other sensitive and multiplex immunoassay methods, which are of interest at present and are focused by our group. The developed IAC-icELISA technique could be a feasible quantitative method for STG in agricultural samples without expensive equipment and a friendly and high-throughput way for STG monitoring.
